# Development of a Frailty Index in the Irish Hip Fracture Database

**DOI:** 10.1007/s00402-022-04644-6

**Published:** 2022-10-09

**Authors:** Mary Walsh, Helena Ferris, Louise Brent, Emer Ahern, Tara Coughlan, Roman Romero-Ortuno

**Affiliations:** 1 School of Public Health, Physiotherapy and Sports Science, University College Dublin (UCD), Dublin, Ireland; 2 Department of Public Health, Health Service Executive-South, Killarney, Ireland; 3 National Office of Clinical Audit (NOCA) and Royal College of Surgeons in Ireland (RCSI), Dublin, Ireland; 4 Cork University Hospital (CUH) and University College Cork (UCC), Cork, Ireland; 5 Discipline of Medical Gerontology, School of Medicine, Trinity College Dublin (TCD), Dublin, Ireland

**Keywords:** Frailty, Frailty Index, Hip fracture, Irish Hip Fracture Database, National clinical audit

## Abstract

**Introduction:**

In older people, hip fracture can lead to adverse outcomes. Frailty, capturing biological age and vulnerability to stressors, can indicate those at higher risk. We derived a frailty index (FI) in the Irish Hip Fracture Database (IHFD) and explored associations with prolonged length of hospital stay (LOS ≥ 30 days), delirium, inpatient mortality and new nursing home admission. We assessed whether the FI predicted those outcomes independently of age, sex and pre-operative American Society of Anaesthesiology (ASA) score.

**Materials and methods:**

A 21-item FI was constructed with 17 dichotomous co-morbidities, three 4-level ordinal pre-morbid functional variables (difficulty with indoor mobility, outdoor mobility, and shopping) and nursing home provenance (yes/no). The FI was computed as the proportion of items present and divided into tertiles (low, medium, high risk). Independent associations between FI and outcomes were explored with logistic regression, from which we extracted adjusted Odds Ratios (aOR) and Areas Under the Curve (AUC).

**Results:**

From 2017 to 2020, the IHFD included 14,615 hip fracture admissions, mean (SD) age 80.4 (8.8), 68.9% women. Complete FI data were available for 12,502 (85.5%). By FI tertile (low to high risk), prolonged LOS proportions were 5.9%, 16.1% and 23.1%; delirium 5.5%, 13.5% and 17.6%; inpatient mortality 0.6%, 3.3% and 10.1%; and new nursing home admission 2.2%, 5.9% and 11.3%. All associations were statistically significant (*p* < 0.001) independently of age and sex. AUC analyses showed that the FI score, added to age, sex, and ASA score, significantly improved the prediction of delirium and new nursing home admission (*p* < 0.05), and especially prolonged LOS and inpatient mortality (*p* < 0.001).

**Conclusions:**

A 21-item FI in the IHFD was a significant predictor of outcomes and added value to traditional risk markers. The utility of a routinely derived FI to more effectively direct limited orthogeriatric resources requires prospective investigation.

**Supplementary Information:**

The online version contains supplementary material available at 10.1007/s00402-022-04644-6.

## Introduction

In older adults, hip fracture is associated with adverse outcomes [1, 2]. Older people living with frailty may be at greatest clinical risk [3, 4]. In Geriatric Medicine, the concept of frailty in an older adult refers to vulnerability to stressors due to dysregulation in multiple physiological systems [5, 6]. Given the marked heterogeneity in the older population, frailty captures biological age more accurately than chronological age [7, 8]. In clinical practice and research, frailty has been measured by various methods [9, 10]. One validated approach is the Frailty Index (FI) based on cumulative health deficits [11], which is particularly suitable in routinely collected health databases [12].

In an older adult, a hip fracture and its subsequent management pose significant physiological stressors. During and around those, older patients benefit from the coordinated efforts of multiple hospital professionals in an area of practice called orthogeriatrics [13, 14]. In Ireland, fully embedded orthogeriatric teams are still lacking in many hospitals [15]. However, routinely collected data could help non-orthogeriatric clinicians detect the presence and severity of frailty so that frailer patients can be identified earlier in the treatment pathway. Previous risk stratification efforts in hip fracture care have considered demographic factors such as age [16] and sex [17]. The American Society of Anaesthesiologist (ASA) score has also been regarded as helpful [18]. However, in other areas of surgical practice, the operationalisation of FIs has shown to add value to traditional risk markers [19, 20]. Therefore, we hypothesised that the development of a FI could help improve risk stratification in the Irish hip fracture population.

The aim of this study was to derive a FI within the Irish Hip Fracture Database (IHFD) and explore its associations with prolonged LOS, development of delirium during the hospital admission, inpatient mortality and new nursing home placement. We also assessed whether the FI was predictive of those outcomes independently of age, sex and ASA score. A FI retrospectively validated from this dedicated national data source aimed to be a first step towards its future prospective clinical validation in Ireland and internationally.

## Methods

### Study design

This was an observational study of national clinical audit data in Ireland.

### Setting and data source

Data from the national IHFD were used. The IHFD is a clinically-led web-based audit that was established in 2012 in collaboration with the Irish Gerontological Society (IGS) and Irish Institute of Trauma and Orthopaedic Surgery (IITOS). The IHFD is operationally managed and governed by the National Office of Clinical Audit (NOCA). Since 2017 the audit has achieved greater than 95% coverage of national hip fracture cases treated across 16 acute hospitals [21]. In 2018, a Best Practice Tariff system was introduced in Ireland with supplementary financial renumeration being provided to trauma services for meeting the clinical, data quality and governance standards for hip fracture care [22].

Full details of the database including results over time have been reported previously [21]. In brief, the dataset includes variables specifically collected to audit hip fracture quality of care, in addition to data collected via the Hospital Inpatient Enquiry (HIPE) electronic system. The HIPE system is the principal source of demographic, clinical and administrative information on all discharges and deaths from publicly funded acute hospitals in Ireland. In the absence of a unique health identifier in Ireland, HIPE data represents single episodes of care within a single clinical site. Co-morbidities are recorded if they were active or relevant during the hospital admission of interest [23].

### Study population

IHFD cases aged 60 years or older with a primary or secondary diagnosis of hip fracture with a discharge date between 1 January 2017 and 31 December 2020 were included. The diagnosis of hip fracture was based on the International Classification of Diseases, Tenth Revision, Australian Modification (ICD-10-AM) diagnosis codes S72.00-S72.2 or a specified type of fracture other than periprosthetic (e.g. intracapsular, intertrochanteric and subtrochanteric).

### Items for the Frailty Index

An FI can be constructed in a health database that contains information on symptoms, signs, morbidities, disabilities and/or even laboratory abnormalities that fulfil criteria for health deficits. A standard FI requires a sufficient number of unweighted deficits (usually 30–70) that cover a range of systems, accumulate with age without saturating too early, and are associated with adverse health outcomes [12]. However, modified FIs with less than 30 items have been reported in the surgical literature [24–26]. Based on previous literature and a recently published scoping review of frailty measurements used in hospitalised older orthopaedic populations [27–30], 15 dichotomous co-morbidities were chosen for inclusion. These were based on the criterion that they should be chronic conditions likely to have preceded the hip fracture event. They also met health deficit requirements in that they accumulate with age and do not saturate too early [12]. The prevalence of these conditions in the IHFD was also validated against that previously found in community-dwelling older adults deemed to be at high risk for fracture in primary care in Ireland [31]. These 15 conditions were: ischemic heart disease, peripheral vascular disease, congestive heart failure, chronic obstructive pulmonary disease, hypertension, diabetes mellitus, dementia, stroke/ hemiplegia, chronic renal disease, cancer, Parkinson’s disease, arthritis, reflux/ peptic ulcer disease, chronic liver disease and atrial fibrillation. ICD-10-AM codes were kept broad where appropriate to allow for maximum sensitivity and were informed by previous international literature [32–34]. Full details of codes are listed in Supplementary Table 1.

Two further deficits included in the FI were: a history of previous fragility fracture and pre-admission pressure ulcer. The former is recorded in the IHFD as yes or no. As regards the latter, pressure ulcers that are developed during the hospital admission are recorded by the IHFD as one of the core quality standards of the audit (i.e. any new pressure ulcer, grade 2 or higher, that develops after admission and within 120 days during that admission) [35]. The presence of a primary or secondary diagnosis of the ICD-10-AM code L89 in the absence of the IHFD recording of new pressure ulcer was used as an indicator of pre-admission pressure ulcer.

The New Mobility Score (NMS) was introduced to the IHFD in 2016 to record patients’ pre-fracture mobility status and has excellent completeness since 2017. It has been found to be valid, reliable and predictive of rehabilitation potential in the older hip fracture population [36–38]. The scale is made up of three sections in which patients self-report level of assistance required with indoor mobility, outdoor mobility and shopping. Each section is scored on a 4-level ordinal scale from ‘unable’ to ‘no difficulty and no aid’. These were recoded as 3 additional items of the FI (where in each of them, higher scores indicated worse pre-morbid mobility, scored as follows: no difficulty and no aid = 0; with a walking aid = 0.33; with help from another person = 0.67; and unable = 1).

Nursing home provenance (yes or no) was included as the last FI deficit. Cases were identified as nursing home residents if they were recorded in the IHFD as having been admitted from or discharged back to a nursing home. While being a resident of a nursing home is not per se a biological marker of frailty, it is a very important proxy of other constructs that are unmeasured within the IHFD, including dependence in self-care activities and cognitive impairment [39].

### Calculation of the Frailty Index

For calculation of the FI, the presence of each of the 21 items (Supplementary Table 1) was summed per patient, and this total summed value was divided by 21 to give a FI score between 0 and 1. The FI was then divided into tertiles of low, medium and high risk.

### Additional characterisation and comparative predictors

For additional characterisation, we collected information on type of fracture (intracapsular, intertrochanteric/ subtrochanteric or other) and type of surgical repair (arthroplasty, internal fixation, other or non-operative). For characterisation and comparative prediction purposes, we also collected age, sex, and the pre-operative ASA score, all of which are available in the IHFD. The ASA score is based on the assessment of a patient’s overall health based on five classes: I: fit and healthy; II: mild systemic disease; III: severe systemic disease that is not incapacitating; IV: incapacitating disease that is a constant threat to life; V: moribund patient who is not expected to live 24 h with or without surgery [40].

### Outcomes

Mortality outcomes included inpatient mortality at 7 and 14 days, as well as the overall inpatient mortality proportion. Prolonged LOS was defined as 30 days or more. Development of delirium during the hospital admission was recorded based on a secondary diagnosis of ICD-10-AM code F05. New institutionalisation is specifically recorded in the IHFD by clinical teams and is defined as new admission to a nursing home or long-term care facility on discharge from the acute hospital.

### Analysis

Statistical analyses were performed with IBM^®^ SPSS^®^ for Windows version 26 (IBM Corp., Armonk, N.Y., USA). Demographic details of all cases were summarised with descriptive statistics (mean and standard deviation, count and percentage).

Multivariable logistic regression was used to explore the association between FI tertiles (with the lowest tertile as reference category) with each dichotomous outcome, whilst adjusting for age (in single years) and sex. Adjusted Odds Ratios (aOR) were extracted with 95% Confidence Intervals (CIs).

For all outcomes, Receiver Operating Curve (ROC) analyses (based on model probability levels saved from the respective regression models) were conducted to compare the predictive ability of two different models: one composed by age, sex and ASA score; and one composed by age, sex, ASA score and the FI score. Areas Under the Curve (AUC) statistics were calculated with 95% CIs. As per rule of thumb from Hosmer and Lemeshow, we considered AUC < 0.7 as poor discrimination; ≥ 0.7 to < 0.8 as fair discrimination; and ≥ 0.8 as good discrimination [41]. Statistical comparisons between two AUCs were conducted with MedCalc^®^ version 20.111 (MedCalc Software, Ostend, Belgium), using the comparison of areas under independent ROC curves command.

Statistical significance was defined as *p* < 0.05 throughout.

### Ethical approval

Ethical approval for analysis of the Irish Hip Fracture Database was obtained from the Royal College of Surgeons in Ireland (REC202001017).

## Results

Based on the IHFD, 16 hospitals discharged 14,615 patients with hip fracture between 1 January 2017 and 31 December 2020. A total of 12,502 (86%) had complete data available for calculation of the FI. The numbers missing data for previous fracture, indoor mobility, outdoor mobility and shopping were *n* = 1010, *n* = 940, *n* = 1120 and *n* = 1178, respectively. Mean age of included patients was 80.4 years (8.8 SD) and 69.6% (*n* = 8695) were female. Table 1 shows patient characteristics by FI tertile (low: ≤ 0.05; medium: ≤ 0.15; high: > 0.15). With increasing FI tertile, there were corresponding increases in mean age, male sex, and higher ASA grades. The most common fracture type across all tertiles was intracapsular, and the most common type of surgical repair was arthroplasty (including hemiarthroplasty and total hip arthroplasty).

**Table 1:** Characteristics of included hip fracture patients by Frailty Index tertile (*n* = 12,502)

	Low tertile *N* = 4830	Medium tertile *N* = 3981	High tertile *N* = 3691
Mean age (SD)	76.9 (8.6)	81.7 (8.4)	83.7 (7.8)
Female sex [*n* (%)]	3453 (71.5)	2788 (70.0)	2454 (66.5)
ASA grade (*n* %)			
I or II	2790 (57.8)	1211 (30.4)	547 (14.8)
III	1593 (33.0)	2203 (55.3)	2264 (61.3)
IV or V	83 (1.7)	226 (5.7)	513 (13.9)
Unknown	364 (7.5)	341 (8.6)	367 (9.9)
Type of fracture (*n* %)			
Intracapsular	2679 (55.5)	1898 (47.7)	1795 (48.6)
Intertrochanteric/Subtrochanteric	1837 (38.0)	1808 (45.4)	1636 (44.3)
Other	314 (6.5)	175 (4.4)	260 (7.0)
Type of surgery (*n* %)			
Arthroplasty	2576 (53.3)	1907 (47.9)	1751 (47.4)
Internal fixation	2038 (42.2)	1839 (46.2)	1676 (45.4)
Other surgery	92 (1.9)	97 (2.4)	74 (2.0)
Non-operative	124 (2.6)	138 (3.5)	190 (5.2)

The associations between FI tertiles and the outcomes considered are summarised in Table 2 along with the proportion of patients in each tertile who experienced each outcome. The proportion of patients who developed delirium, had prolonged LOS, died in hospital or were newly admitted to a nursing home increased by FI tertile. 5.5% of low tertile patients developed delirium compared to 13.5% in the medium tertile and 17.6% in the high tertile. In relation to LOS in the acute hospital setting, 5.9% of those in the low tertile had a prolonged LOS compared to 16.1% in the medium tertile and 23.1% in the high tertile. By increasing FI tertiles, overall inpatient mortality was 0.6%, 3.3% and 10.1%; and new nursing home admission 2.2%, 5.9% and 11.3%. On multivariable logistic regression analyses, all the above associations were statistically significant (*p* < 0.001) independently of age and sex, with increasing aORs by FI tertile (Table 2).

**Table 2:** Association between Frailty Index tertiles and outcomes

	Low tertile *N* = 4830	Medium tertile *N* = 3981			High tertile *N* = 3691		
	N (%)	*N* (%)	aOR (95% CI)	*p* value	*N* (%)	aOR (95% CI)	*p* value
Development of delirium	266 (5.5)	539 (13.5)	2.20 (1.88–2.57)	< 0.001	649 (17.6)	2.74 (2.34–3.20)	< 0.001
Prolonged length of stay (≥ 30 days)	284 (5.9)	639 (16.1)	2.88 (2.48–3.35)	< 0.001	853 (23.1)	4.38 (3.77–5.08)	< 0.001
New nursing home admission*	103 (2.2)	209 (5.9)	2.22 (1.74–2.84)	< 0.001	237 (11.3)	4.38 (3.43–5.59)	< 0.001
7 days inpatient mortality	12 (0.2)	36 (0.9)	2.89 (1.49–5.61)	< 0.001	104 (2.8)	8.30 (4.50–15.32)	< 0.001
14 days inpatient mortality	16 (0.3)	65 (1.6)	4.16 (2.39–7.24)	< 0.001	184 (5.0)	12.13 (7.20–20.45)	< 0.001
Overall inpatient mortality	30 (0.6)	131 (3.3)	4.75 (3.17–7.10)	< 0.001	374 (10.1)	14.74 (10.07–21.59)	< 0.001

Low risk is reference category; ORs adjusted for age in single years and sex

*N = 10,479 not admitted from nursing home who had complete data for frailty index calculation

Table 3 shows AUC values and 95% CIs for two models: Model 1 with age, sex and ASA score; and Model 2 with the addition of the FI score. The highest AUC was for inpatient mortality, Model 2 (AUC 0.81). Statistical comparison of AUCs between Models 1 and 2 showed that the FI score, added to age, sex and ASA score, added significant value to the prediction of delirium and new nursing home admission (*p* < 0.05), and especially to prolonged LOS and inpatient mortality (*p* < 0.001).

**Table 3:** Areas under the curve comparisons

	Delirium	Prolonged LOS	Inpatient death	New nursing home admission
Model 1: Age, sex and ASA	0.66 (0.65–0.68)	0.64 (0.62–0.65)	0.75 (0.73–0.77)	0.64 (0.62–0.66)
Model 2: Age, sex, ASA + FI	0.69 (0.67–0.70)	0.70 (0.68–0.71)	0.81 (0.79–0.83)	0.67 (0.65–0.69)
Model 1 vs 2: *p* value for statistical comparison of AUCs	*p* = 0.011	*p* < 0.001	*p* < 0.001	*p* = 0.047

## Discussion

This study derived a FI within the IHFD and explored associations with prolonged LOS, delirium, inpatient mortality and new admission to a nursing home. In 12,502 cases with hip fracture between 2017 and 2020, we found that all associations were statistically significant independently of age and sex. Moreover, AUC statistical analyses showed that the FI score, added to age, sex and ASA score, significantly improved the prediction of delirium and new nursing home admission (*p* < 0.05), and especially prolonged LOS and inpatient mortality (*p* < 0.001). Specifically, the FI combined with age, sex and ASA grade had good discrimination for predicting inpatient death and fair discrimination for prolonged LOS, delirium and new admission to a nursing home.

Our findings resonate with those obtained by other groups internationally utilising FIs. For example, employing a modified 33-item FI, Shen et al. showed added value for the prediction of postoperative complications in Chinese older patients with hip fracture [42]. In the American College of Surgeons National Surgical Quality Improvement Program (NSQIP) database, Traven et al. showed that a modified FI of only 5 items (diabetic status, history of chronic obstructive pulmonary disease or current pneumonia, congestive heart failure, hypertension requiring medication, and non-independent functional status) was independently predictive of postoperative morbidity and mortality in older hip fracture patients [43]. Bellamy et al. also analysed the NSQIP database and showed positive associations between an 11-item FI and a range of adverse hospital outcomes [44]. However, in the 2013 National Inpatient Sample, Ondeck et al. showed that after surgical management of hip fractures, a modified 11-item FI was not superior to a 29-item Elixhauser’s Comorbidity Measure in predicting the occurrence of in-hospital adverse outcomes [45]. The inclusion of relevant co-morbidities may be important, so in our 21-item FI, 17 deficits were co-morbidities, to which we added three functional performance items and nursing home provenance. In relation to previous FIs, strengths of our FI are its derivation from a national multicentric database including a large number of patients, and an intermediate number of comorbidity items making it reasonably comprehensive but not too time consuming. A weakness is its retrospective validation.

Also in keeping with previous literature, higher frailty levels were associated with higher risk of developing delirium during the inpatient stay [46, 47]. Raising awareness of this fact amongst non-specialist clinicians could help trigger interventions (such as proactive identification and management) to minimise the severity and duration of delirium, which is known to prolong hospital LOS and lead to adverse patient outcomes including new institutionalisation and mortality [48, 49]. Easy-to-use risk identification strategies that can be proactively implemented by non-specialists are important because, with a mean LOS of 17 days for Irish hip fracture patients [22], there is a growing need to prioritise time spent in the acute hospital setting, where possible, to the immediate perioperative period and maximise the use of early supported discharge options and community-based services for the rehabilitation phase [50].

The IHFD is a large national dataset of older adults with excellent data coverage and completeness. It provides a reliable infrastructure for the collection of high-quality data, which are measured against explicit national standards of care, namely the Irish Hip Fracture Standards [51]. The IHFD contains data pertaining to > 25,000 hip fracture cases since its inception in 2012. However, our study included data from 2017 onwards as data coverage in the earlier years of the database was suboptimal. Other limitations of our study are its retrospective, observational design and the fact that it is based on routinely collected data. Our comorbidity data was derived from the HIPE system, which is an important information source for research and health service planning activities in Ireland but not exempt of limitations [52]. Whilst in the present study we followed the standard methodology for the derivation of the FI [12], in future research weight estimates for each FI variable could be explored. The utility of a routinely derived FI to more effectively direct limited orthogeriatric and rehabilitative resources requires further investigation with a prospective research design.

The analysis outlined in this paper demonstrated the value of synthesising routinely collected data to generate a FI for the purpose of risk stratification in Irish hip fracture patients. A 21-item FI in the IHFD was a significant predictor of adverse outcomes post-hip fracture and added value to traditional risk markers such as age, sex and ASA score; yet predictions by age, sex and ASA alone were not insignificant. Based on our findings, we advocate for an integrated, proactive risk-stratification approach that considers all the risk markers available, rather than favouring one single risk identification tool over another. In the absence in many Irish hospitals of inpatient orthogeriatric teams [15], an easy to implement approach guided by demographics, anaesthetic information, comorbidities, functional status and nursing home provenance may help non-orthogeriatric clinicians identify the most vulnerable patients early in the care pathway [53].

## Conclusion

A retrospectively validated FI in the IHFD was predictive of adverse outcomes independently of age, sex and ASA score. This FI requires future prospective clinical validation in Ireland and internationally.

## Supplementary Information

Below is the link to the electronic supplementary material.

Supplementary file1 (DOCX 41 KB)
