# Supplementary material for: Development of a Frailty Index in the Irish Hip Fracture Database
Source: Arch Orthop Trauma Surg. 2022 Oct 9;143(7):4447–54. doi: 10.1007/s00402-022-04644-6 (PMC10293399; doi:10.1007/s00402-022-04644-6)
Supplement: Supplementary file 1 — Supplementary file1 (DOCX 41 KB) [file 402_2022_4644_MOESM1_ESM.docx]

**Supplementary Table 1. The 21-item IHFD Frailty Index.**

| **Frailty Index items** | **Scoring** | **ICD-10-AM** |
| --- | --- | --- |
| 1. Ischemic heart disease | No = 0  Yes = 1 | I20 I21 I22 I23 I24 I25 |
| 1. Peripheral vascular disease | No = 0  Yes = 1 | I70 I71 I731 I738 I739 I771 I790 I791 I798 K551 K558 K559 Z958 Z959 R02 I96 |
| 1. Congestive heart failure | No = 0  Yes = 1 | I110 I130 I132 I255 I420 I425 I426 I427 I428 I429 I43 I50 J81 |
| 1. Chronic obstructive pulmonary disease | No = 0  Yes = 1 | J40 J41 J42 J43 J44 J45 J46 J47 J60 J61 J62 J63 J64 J65 J66 J67 J684 J701 J703 |
| 1. Hypertension | No = 0  Yes = 1 | I10 I11 I12 I13 I15 |
| 1. Diabetes mellitus | No = 0  Yes = 1 | E09 E10 E11 E13 E14 |
| 1. Dementia | No = 0  Yes = 1 | F01 F02 F03 F04 F061 F068 G132 G138 G30 G310 G311 G312 G914 G94 R4181 |
| 1. Stroke/ hemiplegia | No = 0  Yes = 1 | G45 G46 H340 H341 H342 I60 I61 I62 I63 I64 I65 I66 I67 I68 I69 G81 |
| 1. Chronic renal disease | No = 0  Yes = 1 | N03 N05 N18 Z940 I129 I130 I1310 I120 I1311 I132 N19 N25 Z49 Z992 |
| 1. Cancer | No = 0  Yes = 1 | C00 C01 C02 C03 C04 C05 C06 C07 C08 C09 C10 C11 C12 C13 C14 C15 C16 C17 C18 C19 C20 C21 C22 C23 C24 C25 C26 C30 C31 C32 C33 C34 C37 C38 C39 C40 C41 C43 C44 C45 C46 C47 C48 C49 C50 C51 C52 C53 C54 C55 C56 C57 C58 C60 C61 C62 C63 C64 C65 C66 C67 C68 C69 C70 C71 C72 C73 C74 C75 C76 C77 C78 C79 C80 C81 C82 C83 C84 C85 C86 C88 C90 C91 C92 C93 C94 C95 C96 C97 D00 D01 D02 D03 D04 D05 D06 D07 D09 |
| 1. Parkinson’s disease | No = 0  Yes = 1 | G20 G21 G22 |
| 1. Arthritis | No = 0  Yes = 1 | M05 M06 M07 M08 M09 M1 M32 M34 M353 M315 M33 M351 M360 |
| 1. Reflux/ peptic ulcer disease | No = 0  Yes = 1 | K2 K30 K31 |
| 1. Chronic liver disease | No = 0  Yes = 1 | I85 I98 K65 K67 K7 K93 R18 K702 K703 K73 K717 K740 K742 K746 K743 K744 K745 I120 I1311 I132 N185 N186 N19 N25 Z49 Z992 |
| 1. Atrial fibrillation | No = 0  Yes = 1 | I48 |
| 1. Previous fragility fracture | No = 0  Yes = 1 |  |
| 1. Pre-admission pressure ulcer | No = 0  Yes = 1 | L89 in the absence of IHFD recording  of new in-hospital pressure ulcer |
| 1. Indoor mobility | No difficulty and no aid = 0; with a walking aid = 0.33; with help from another person = 0.67; unable = 1 | |
| 1. Outdoor mobility | No difficulty and no aid = 0; with a walking aid = 0.33; with help from another person = 0.67; unable = 1 | |
| 1. Shopping | No difficulty and no aid = 0; with a walking aid = 0.33; with help from another person = 0.67; unable = 1 | |
| 1. Nursing home resident pre-fracture | No = 0  Yes = 1 |  |
